# Sterile Inflammatory Response and Surgery-Related Trauma in Elderly Patients with Subtrochanteric Fractures

**DOI:** 10.3390/biomedicines12020354

**Published:** 2024-02-02

**Authors:** Flaviu Moldovan

**Affiliations:** Orthopedics—Traumatology Department, Faculty of Medicine, “George Emil Palade” University of Medicine, Pharmacy, Science, and Technology of Targu Mures, 540142 Targu Mures, Romania; flaviu.moldovan@umfst.ro; Tel.: +40-754-671-886

**Keywords:** subtrochanteric femur fractures, long Gamma Nail, reduction technique, sterile inflammation, hematologic biomarkers

## Abstract

Sterile inflammation is a natural response of the organism in the absence of microorganisms, which is triggered in correspondence with the degree of tissue damage sustained after a surgical procedure. The objective of this study was to explore the values of postoperative hematological-derived biomarkers in assessing the sterile inflammatory response magnitude related to the invasiveness of the surgical reduction technique used for subtrochanteric fractures (STFs) treatment. A retrospective, observational cohort research was conducted between January 2021 and October 2023 that included a total of 143 patients diagnosed with acute subtrochanteric fractures who underwent long Gamma Nail (LGN) fixation. According to the surgical reduction technique used, they were divided into two groups: group 1, which consisted of those with a closed reduction and internal fixation (CRIF); and group 2, which consisted of those with an open reduction internal fixation (ORIF). Between groups, statistically significant differences (*p* < 0.05) were found in relation to days to surgery, length of hospital stay (LOHS), duration of surgery, postoperative hemoglobin (HGB) levels, neutrophil–lymphocyte ratio (NLR), platelet–lymphocyte ratio (PLR), monocyte–lymphocyte ratio (MLR), systemic inflammation index (SII), systemic inflammation response index (SIRI), and aggregate inflammation systemic index (AISI). The receiver operating characteristics (ROC) curve analysis revealed that all ratios presented a high diagnostic ability (*p* < 0.0001) with NLR > 6.95 being the most reliable (sensitivity 94.8% and specificity 70.6%). Moreover, the multivariate regression model confirmed that sterile immune response after orthopedic interventions can be assessed in an almost equal and non-dependent manner using these biomarkers. Postoperative NLR, PLR, MLR, SII, SIRI, and AISI ratios are closely correlated to the sterile inflammatory response magnitude, due to the extent of surgical dissection performed during internal fixation procedures of subtrochanteric femur fractures.

## 1. Introduction

Subtrochanteric fractures (STFs) are common among the elderly and constitute a significant cause of mortality and increased morbidity [1,2]. Despite various treatment options, closed reduction and internal fixation (CRIF) with intramedullary nails has become the predominant approach [3,4,5]. Surgical decisions, including technique, duration, and blood loss, are influenced by factors such as fracture characteristics, patient body type, surgeon experience, and technical skill [6]. While a minimally invasive approach is preferable for reduced complications and a faster recovery, achieving this outcome is not always feasible in practice. The primary surgical goal remains achieving a stable and precise fracture reduction, favoring CRIF when possible [7,8].

Sterile inflammation represents a term that describes a complex systemic inflammatory process, triggered by tissue damage during surgery [9]. As the name suggests, it is considered a non-infectious, damage-associated molecular pattern (DAMP) response that can further contribute to cardiometabolic disease, as Cho et al. [10] demonstrated. Lead mediators of this process include leukocytes that are present due to altered tissue and platelets (PLTs) that activate and aggregate at vascular injury sites [11]. In these dynamics, neutrophils are known to be the most abundant leukocytes constituting the first line of defense [12] and have a role in fracture healing by producing fibronectin^+^ in the extracellular matrix [13]. Monocytes have high infiltrative properties that can aid the removal pathogen-associated molecular patterns (PAMPs) and cellular debris through phagocytosis [14]. Lymphocytopenia can predict mortality and was associated with major postoperative complications in multiple studies [15,16,17].

More recently, this knowledge was used in various medical fields to compute novel biomarkers that can quantify inflammation and identify predictive patterns. For example, Lijuan et al. [18] investigated rheumatoid arthritis progression in relation to neutrophil–lymphocyte ratio (NLR), platelet–lymphocyte ratio (PLR) and monocyte-to-lymphocyte ratio (MLR). Various complications can be assessed using these ratios, including sepsis after procedures like percutaneous nephrolithotomies [19]. Combined ratios, such as the aggregate index of systemic inflammation (AISI), the systemic immune-inflammation index (SII), and the systemic inflammation response index (SIRI) have proven their use in COVID-19 patients who had to be admitted to intensive care units [19,20]. A use for these markers can be also seen in trauma care, as Wang et al. [21] established a severity prediction in isolated tibial plateau fractures by using blood-derived NLR. A recent study performed by Zhou et al. [22] demonstrated a similar result, where they added PLR and SII, in determining the severity and prognosis of acute spinal cord injuries.

Up until the present time, the extent of the soft tissue dissection during surgical interventions and the possible implications of the sterile inflammatory response have not yet been addressed. Thus, the purpose of this study was to establish a correlation between postoperative NLR, PLR, MLR, SII, AISI, and SIRI ratios and the sterile inflammatory response magnitude due to the invasiveness of the reduction technique used in subtrochanteric femur fracture internal fixation.

## 2. Materials and Methods

### 2.1. Study Design and Participants

**Figure 1:**
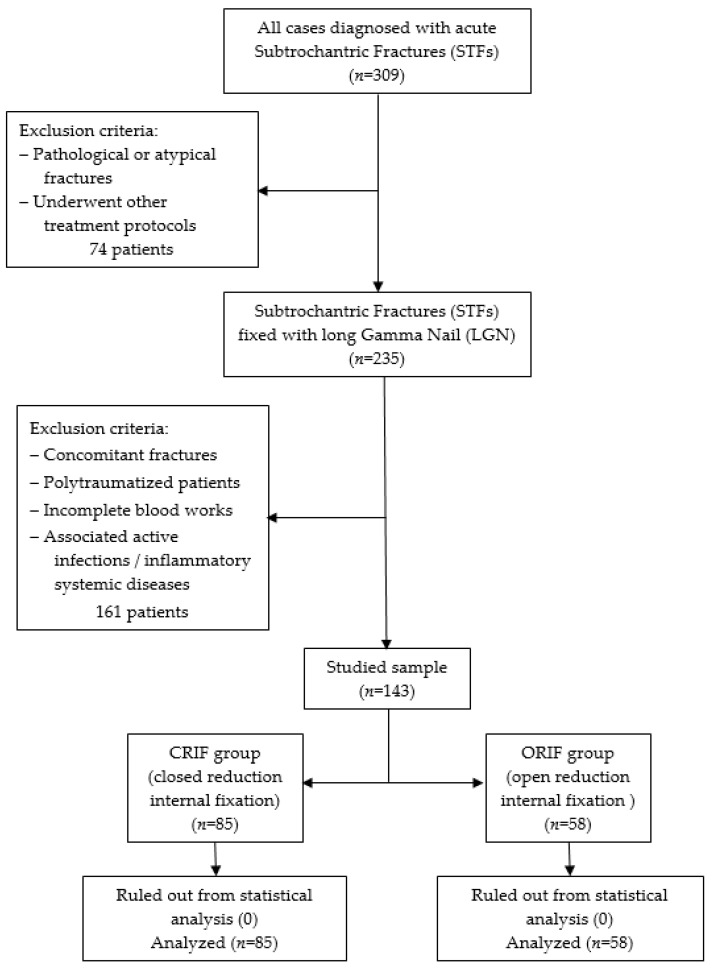
Flow chart for the studied sample selection.

A retrospective, observational cohort research was conducted at the Orthopedics-Traumatology Department of the County Emergency Clinical Hospital of Targu Mures, Romania between January 2021 and October 2023. Inclusion criteria consisted of patients with acute subtrochanteric fractures who underwent long Gamma Nail (LGN) fixation as treatment. Those who presented the following were excluded: underwent other treatment protocols (fixed angled plates with a 95-degree blade plate or condylar screw); old fractures; pathological and atypical fractures related to Denosumab or bisphosphonate use; concomitant fractures; polytraumatized patients; incomplete blood work; associated active infection; or inflammatory systemic disease. Thus, two groups of participants were formed depending on the reduction technique used: those treated with CRIF (closed reduction internal fixation, *n* = 85), corresponding to low surgical-related trauma, and those treated with ORIF (open reduction internal fixation, *n* = 58), corresponding to high surgical-related trauma. A detailed overview of the studied sample is provided in Figure 1.

### 2.2. Data Acquisition

With the use of the hospital’s digital database, the following information was collected: (1) Age, gender, living area; (2) Risk factors associated with lifestyle, such as smoking, alcohol use, and obesity (BMI ≥ 30); (3) Medical comorbidities (of Senile dementia—SD, Essential hypertension—EH, Atrial fibrillation—AF, Chronic ischemic heart disease—CIHD, Peripheral venous insufficiency—PVI, Pulmonary fibrosis—PF, Chronic obstructive pulmonary disease—COPD, Dyslipidemia, Diabetes); (4) Surgical factors, such as side of the fracture, American Society of Anesthesiologists (ASA) score, type of anesthesia, days to surgery, length of hospital stay (LOHS), and duration of the surgical intervention (minutes); (5) laboratory data at admission and on the first day after surgery, which included: neutrophils count, lymphocyte count, monocyte count, platelet (PLT) count, aspartate–transaminase (AST)/alanine–transaminase (ALT) ratio, white blood cell count (WBC), red blood cell count (RBC), and hemoglobin level.

### 2.3. Hematologic Derived Markers of Inflammation

For the next stage of the study, six inflammatory markers derived from laboratory blood work were computed to investigate their relation to the invasiveness of the reduction technique of the proposed surgical procedure.

The first three markers were defined as: (1) the neutrophil–lymphocyte ratio (NLR), which is the division of the neutrophile count and the lymphocyte count; (2) the platelet–lymphocyte ratio (PLR), which is the division of the platelet count and the lymphocyte count; (3) the monocyte–lymphocyte ratio (MLR), which is the division of the monocyte count and the lymphocyte count.

1. $\mathrm{NLR}=\frac{Neutrophile\;count}{Lymphocyte\;count}$
2. $PLR=\frac{Platelet\;count}{Lymphocyte\;count}$
3. $MLR=\frac{Monocyte\;count}{Lymphocyte\;count}$

The next three markers were more complex in analyzing systemic inflammatory changes: (4) the systemic inflammation index (SII), which is the product of the neutrophil count and platelet count divided by the lymphocyte count; (5) the systemic inflammation response index (SIRI), which is the product of the monocyte count and the platelet count divided by the lymphocyte count; (6) the aggregate inflammation systemic index (AISI), which is the product of the neutrophile count, the monocyte count, and the platelet count divided by the lymphocyte count.

4. $SII=\frac{Neutrophile\;count\;\times \;Platelet\;count}{Lymphocyte\;count}$
5. $SIRI=\frac{Monocyte\;count\;\times \;Platelet\;count}{Lymphocyte\;count}$
6. $AISI=\frac{Neutrophile\;count\;\times \;Monocyte\;count\;\times \;Platelet\;count}{Lymphocyte\;count}$

### 2.4. Surgical Procedure

**Figure 2:**
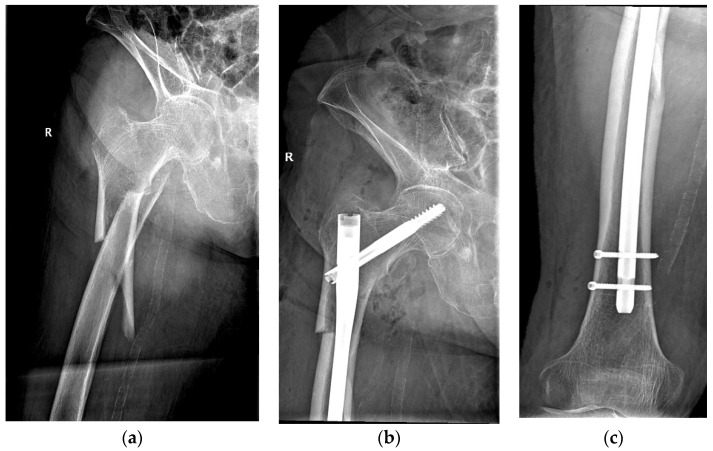
An 84-year-old patient diagnosed with a subtrochanteric fracture after a fall from the same height treated with CRIF with long Gamma Nail fixation: (**a**) preoperative radiograph; (**b**,**c**) postoperative radiographs.

The same surgical technique was performed in all cases by experienced orthopedic surgeons from the department. Patients underwent spinal anesthesia (with 0.5% heavy Marcaine and Sufentanyl) or general anesthesia (with Ketamine and Esmeron) and were also given standard antibioprophylaxis (with Cefuroxime 1.5 g for three days). The long Stryker Gamma3 Nailing System (Stryker Corp., Kalamazoo, MI, USA) was used for the internal fixation under intraoperative fluoroscopy. A dedicated orthopedic table provided closed fracture reduction with an estimated length of incision of 4 to 5 cm, although conversion to open reduction was needed in more demanding cases. Figure 2 shows the pre- and postoperative radiographs of a representative case.

### 2.5. Statistical Analysis

First, a normality check was performed on the categorical variables using the Kolmogorov–Smirnov test, after which they were analyzed using the Student’s *t* test or the Wilcoxon rank sum test. To assess the significant intergroup differences between the categorical variables, a Fisher exact test or Chi-square test were used. A receiver operating characteristic (ROC) curve analysis was performed to identify the diagnostic ability and the cut-off values, based on Youden’s index (Youden index = sensitivity + specificity − 1, with a range from 0–1) for biological indexes and valid variables [23,24]. Furthermore, to determine the independent factors associated with intraoperative invasiveness of the reduction technique, a multivariate logistic regression analysis was conducted, which included postoperative markers and surgical-related measurements [25]. The model was shown to be acceptable using the Hosmer–Lemeshow test with a *p*-value above 0.05. The methodology applied has been previously utilized by Wang et al. [26]. SPSS for Widows, version 29.0.1 (SPSS, Inc., Chicago, IL, USA) was used for the statistical analysis.

## 3. Results

This study included 143 patients (38.5% males) with a mean age of 74 years who were diagnosed with traumatic subtrochanteric fractures and underwent osteosynthesis with long GN implants. Two groups were formed based on the reduction technique used: a closed reduction (CRIF) group with 85 patients (59.44%) and an open reduction (ORIF) group with 58 patients (40.56%).

Then, a ROC curve analysis was used to identify the cut-off points (Table 1) of the pre- and postoperative inflammatory markers (NLR, MLR, PLR, SII, SIRI, and AISI) and of the time-related surgical factors, such as days to surgery, duration of surgery, and LOHS.

**Figure 3:**
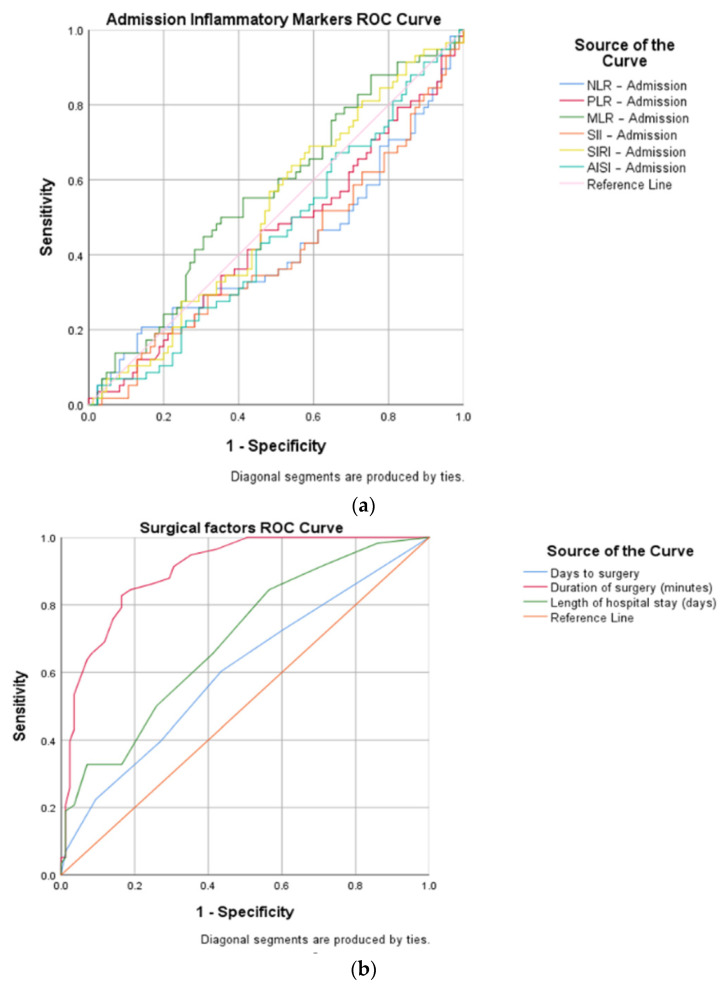

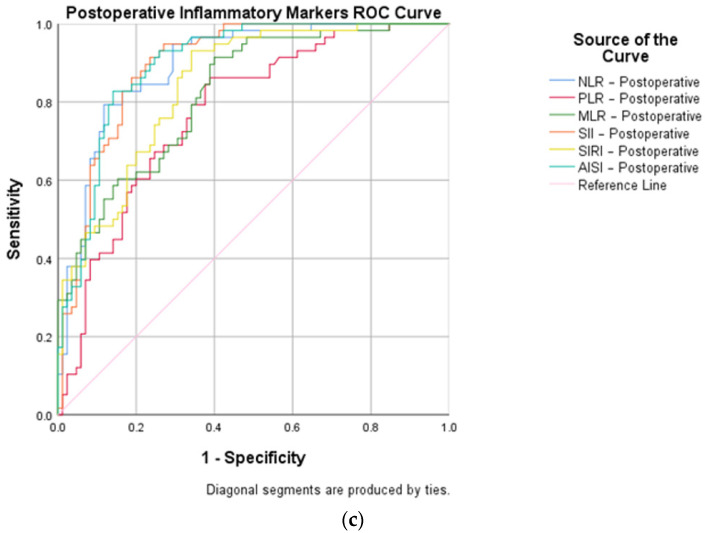
ROC curve representations for open versus closed reduced STFs, concerning: (**a**) inflammatory markers at admission; (**b**) surgical factors (days to surgery, duration of surgery, and length of hospital stay); and (**c**) inflammatory markers on the first day after surgery.

AUC (area under the curve), sensitivity, and specificity (Figure 3) demonstrated a high diagnostic ability of the following variables: postoperative NLR (cut-off 6.95, AUC 0.026, sensitivity 94.8%, specificity 70.6%), postoperative PLR (cut-off 186.13, AUC 0.039, sensitivity 79.3%, specificity 65.9%), postoperative MLR (cut-off 0.66, AUC 0.034, sensitivity 89.7%, specificity 61.2%), postoperative SII (cut-off 1970.47, AUC 0.026, sensitivity 86.2%, specificity 81.2%), postoperative SIRI (cut-off 167.54, AUC 0.032, sensitivity 87.9%, specificity 68.2%), postoperative AISI (cut-off 1857.45, AUC 0.025, sensitivity 82.8%, specificity 85.9%), days to surgery (cut-off 2, AUC 0.049, sensitivity 60.3%, specificity 56.5%), duration of surgery (cut-off 58, AUC 0.025, sensitivity 82.8%, specificity 83.5%), and LOHS (cut-off 8, AUC 0.044, sensitivity 65.5%, specificity 58.8%).

A univariate analysis of the two groups (Table 2) confirmed the statistical difference between the surgical factors related to time: days to surgery (*p* = 0.048), duration of surgery (*p* = 0.004), and LOHS (*p* < 0.0001). Furthermore, the laboratory data after surgery were also significant: neutrophil count (*p* < 0.0001), lymphocyte count (*p* < 0.0001), monocyte count (*p* < 0.0001), PLT count (*p* = 0.008), WBC (*p* < 0.0001), RBC (*p* = 0.002), and HGB (*p* = 0.004). These findings were reflected in the six postoperative markers studied with a *p*-Value < 0.0001.

**Figure 4:**
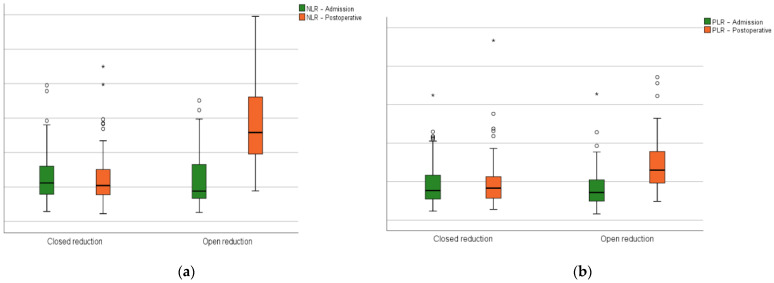

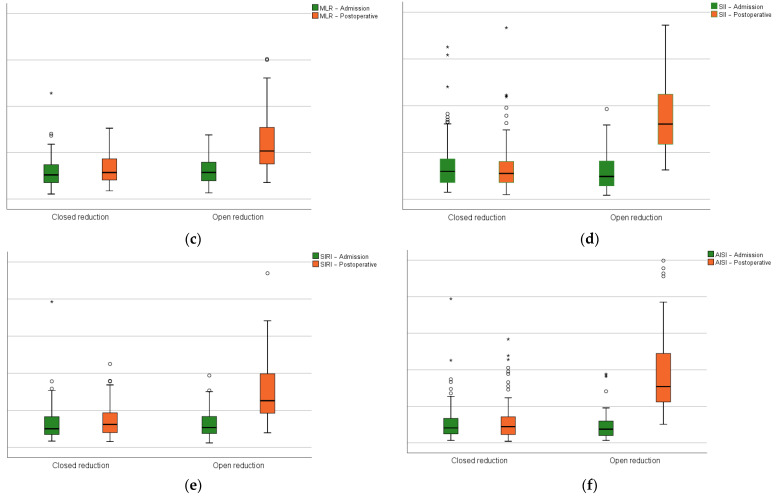
Closed versus open reduction groups boxplots at admission and on the first postoperative day: (**a**) the neutrophil-lymphocyte ratio; (**b**) platelet–lymphocyte ratio; (**c**) monocyte–lymphocyte ratio; (**d**) systemic inflammation index; (**e**) systemic inflammation response index; (**f**) aggregate inflammation systemic index. Circles represent the mild outliers and asterisks represent the extreme outliers.

In relation to the preoperative phase, the hematologic-derived indexes (NLR, MLR, PLR, SII, SIRI, and AISI) had a significant growth after surgery in the open reduction group (Figure 4).

A multivariate model was constructed with the relevant variables identified (Table 3). The goodness of fit was confirmed by the Hosmer–Lemeshow test (X^2^ = 4.564, *p* = 0.813, and Nagelkerke R^2^ = 0.754). The intrinsic immune response triggered by the invasiveness of the surgical technique was suggestive as a whole through the values obtained in the proposed equation: NLR (OR 2.91, 95% CI 1.28–6.61, *p* < 0.0001), PLR (OR 1.59, 95% CI 1.34–1.88, *p* = 0.003), MLR (OR 1.45, 95% CI 1.18–1.78, *p* = 0.005), SII (OR 1.02, 95% CI 1.001–1.03, *p* = 0.039), SIRI (OR 1.04, 95% CI 1.01–1.07, *p* = 0.028), and AISI (OR 1.25, 95% CI 1.05–1.49, *p* = 0.020).

## 4. Discussion

As reported in the recent scientific literature, there seems to be an expanding role in the field of Orthopedics and Traumatology for these inflammatory markers in predicting mortality after hip fracture surgeries [27,28], determination of postoperative pain after arthroplasties [29], and comparing the invasiveness of two type of implants [26,30]. The present research analyzes through the proposed indexes the implications of a commonly met situation during non-elective orthopedic interventions, where the fracture site needs to be exposed to perform a qualitative reduction. All postoperative parameters presented a high diagnostic ability (*p* < 0.0001) with NLR > 6.95 being the most reliable parameter (sensitivity 94.8% and specificity 70.6%). Previous results also suggested that it represents a more robust biomarker as it balances both aspects of immunity: the adaptive component together with acute and chronic inflammation [31,32]. Furthermore, the multivariate regression model confirmed that the sterile immune response after a surgical intervention can be assessed in an almost equal and non-dependent manner using NLR, MLR, PLR, SII, SIRI, or AISI.

In the late 1980s, a new implant, the Gamma Nail, was developed for intertrochanteric hip fracture fixation [33,34]. It yielded the benefits of a rigid and secure fixation that allows early mobilization and assured minimally invasive exposures with a decreased operation time and blood losses [35]. The indications were extended to subtrochanteric, trochantero-diaphyseal and saft fractures of the femur in 1992 by introducing the LGN [31]. Throughout the years, this osteosynthesis material has suffered design modifications, such as exchanges to anodized titanium-based alloys and reductions of the radius curvature to 150 cm to match the natural femoral bowing and decrease the anterior cortex penetration [36].

In this study, the mean age was 73.71 ± 14.34, which confirms the tendency of elderly patients to sustain this fracture due to low traumatic mechanisms, such as falls from same height [2]. Nevertheless, it is important to acknowledge that the magnitude the reduction technique employed rose not only from anatomical considerations but also from the complexity of the fracture, which can be met in the younger population involved in high-energy traumatic events [37]. In terms of etiology, patients on chronic medication with Denosumab or bisphosphonates, especially Alendronate, have been linked with pathological or atypical subtrochanteric fractures due to severe bone turnover suppression [38]. In order not to influence the results of the study, this group was excluded, as these drugs have an overall anti-inflammatory effect that inhibits osteoclast activation and serum levels of tumor necrosis factor (TNF), interleukin (IL)-1, and IL-6 [39].

Many studies have proposed different intra- and perioperative parameters with the scope of comparing two proposed protocols of intervention. For instance, Luo et al. [40] compared laparoscopic versus open surgery in colorectal cancer, using blood loss, length of hospital stays, operative time, and ASA score as variables. In the present study, days to surgery (*p* = 0.048), duration of surgery (*p* < 0.0001), and LOHS (*p* = 0.004) were all surgical measurements that were positively associated with the aggression sustained by the organism during the reduction technique of the STFs. An indirect estimation of higher blood loss could be made between the two groups due to the significant hemoglobin level drop postoperatively (*p* = 0.004) that necessitated transfusions, which is in concordance with results presented by Panteli et al. [41].

Tissue damage without primary wound infection is defined as ‘sterile trauma’ and is present during open surgical procedures. If no exposure to microbes occurs during this initial sterile trauma, an inflammatory process takes place, expanding the term to ‘sterile inflammation’ [42]. During this process, complex plasma biomarker changes occur, such as elevated levels of IL-4, IL-6, and IL-10, and decreased levels of IL-2, IL-12, and Interferon (IFN)-γ [43]. More specifically, after total knee arthroplasties (TKA), immunoglobulins IgA, IgG, and IgM decreased even after three days according to Munoz et al. [44].

A clear consensus between sterile inflammation and systemic inflammatory response syndrome (SIRS) lacks until the present time. Genetic variations and preexisting comorbidities can alter the inflammatory phenotype, thus similar sterile traumas can trigger different responses. What is certain is that cytokines and biochemical parameters, such as C-reactive protein (CRP), which is synthesized due to IL-6 [45], and lower levels of albumin [46] have an essential role in diagnosis. Recently, blood-derived markers were studied by Yahsi et al. [47] who determined that after ureteroscopies NLR acts as an independent risk factor for SIRS development, whereas SII is strongly linked with it. Also, in the field of urology, Kriplani et al. [18] confirmed and added the correlation of the PLR and LMR indices to this reaction. The present study capitalized on these valuable findings and demonstrated a high sensitivity for postoperative ratios in relation to the level of non-infectious systemic inflammatory responses. This may aid financial- and time-related benefits as complete blood works are easily accessible and routinely performed, in contrast to the above-mentioned cytokines, immunoglobulins, or other suggested biochemical determinations.

A set of limitations can be highlighted from this study. Firstly, it is not clear if the proposed markers can distinguish sterile inflammation/SIRS from sepsis using their cut-off values, as open reduction of STFs has been linked with a higher probability of superficial infection and tough deep infection, and non-union rates remain controversial [41]. Furthermore, while these parameters can quantify the sterile inflammatory response to surgical trauma, this study cannot demonstrate the consequences of complications, morbidity, and mortality resulting from an increased sterile inflammatory response. Secondly, the state of the patient prior to surgery needs to be also taken into consideration when determining these indexes, as dehydration may cause hemoconcentration and temporary increases in WBCs. Thirdly, the dynamics of the inflammatory response could have been better assessed through repeated measurements during the hospitalization period, enhancing the results. Lastly, the monocentric retrospective design of this study could be expanded and improved by conducting a multicentric prospective study.

## 5. Conclusions

This is the first study to explore the immediate sterile inflammatory response by demonstrating a clear correlation (*p* < 0.0001) between the postoperative NLR, PLR, MLR, SII, SIRI, and AISI ratios and the extent of surgical dissection performed during intramedullary fixation procedures of STFs. This valuable information can aid orthopedic surgeons with their decision to open the fracture site intraoperatively or continue with the closed reduction techniques, considering factors such as specific surgical timeframes, the particularity of the case, and the overall individualized patient assessment. Furthermore, complex cases of STFs that are prone to an open reduction need longer operative times (*p* < 0.0001), hospital stays (*p* = 0.004), and days to surgery to optimally perform (*p* = 0.048). These parameters and their cut-offs effectively quantify the sterile inflammatory response, identifying the extent of surgical trauma along with procedure duration and blood loss. However, this study does not explore the consequences of a potential increase in sterile inflammatory response on postoperative complications, heightened morbidity, and mortality. Future prospective studies are essential to determine the prognostic and therapeutic utility of these parameters. The current orthopedic clinical practice could benefit from these cost- and time-effective markers, as they can determine indications for treatment protocols and stratify traumatic patients at risk.

## Figures and Tables

**Table 1:** Optimal cut-off values, AUC, and the accuracy prediction identified by the ROC curve analysis.

Variables	Cut-Off Values	AUC	95% CI	Sensitivity	Specificity	*p*-Value
NLR—Admission	6.57	0.421	0.322–0.520	31%	65.9%	0.109
PLR—Admission	156.76	0.451	0.354–0.548	46.6%	54.1%	0.325
MLR—Admission	0.54	0.561	0.466–0.657	55.2%	58.8%	0.214
SII—Admission	1353.31	0.409	0.313–0.505	34.5%	56.5%	0.065
SIRI—Admission	91.83	0.514	0.419–0.610	62.1%	47.1%	0.774
AISI—Admission	834.86	0.456	0.361–0.552	44.8%	0.51%	0.377
NLR—Postoperative	6.95	0.026	0.844–0.947	94.8%	70.6%	<0.0001
PLR—Postoperative	186.13	0.039	0.698–0.851	79.3%	65.9%	<0.0001
MLR—Postoperative	0.66	0.034	0.755–0.889	89.7%	61.2%	<0.0001
SII—Postoperative	1970.47	0.026	0.846–0.947	86.2%	81.2%	<0.0001
SIRI—Postoperative	167.54	0.032	0.781–0.906	87.9%	68.2%	<0.0001
AISI—Postoperative	1857.45	0.025	0.849–0.948	82.8%	85.9%	<0.0001
Days to days	2	0.049	0.509–0.699	60.3%	56.5%	0.035
Duration of surgery (minutes)	58	0.025	0.855–0.951	82.8%	83.5%	<0.0001
Length of hospital stay (days)	8	0.044	0.610–0.783	65.5%	58.8%	<0.0001

Abbreviations: ROC—receiver operating characteristic; AUC—area under the curve; CI—confidence interval; NLR—neutrophil-lymphocyte ratio; PLR—platelet-lymphocyte ratio; MLR—monocyte-lymphocyte ratio; SII—systemic inflammation index; SIRI—systemic inflammatory response index; AISI—aggregate inflammatory systemic index. *p*-value < 0.05 was attributed as statistically significant.

**Table 2:** Univariate analysis of the variables between the two types of reduction techniques.

Variable	All Patients (*n* = 143)	Closed Reduction Group (*n* = 85)	Open Reduction Group (*n* = 58)	*p*-Value
Baseline characteristics				
Age (years), mean ± SD	73.71 ± 14.34	73.27 ± 14.30	74.34 ± 14.49	0.662
Sex, *n* (%) Male Female	55 (38.5) 88 (61.5)	31 (36.5) 54 (63.5)	24 (41.4) 34 (58.6)	0.554
Alcohol (yes), *n* (%)	34 (23.8)	20 (23.5)	14 (24.1)	0.933
Smoking (yes), *n* (%)	35 (24.5)	23 (27.1)	12 (20.7)	0.384
Obesity (yes), *n* (%)	73 (51.0)	40 (47.1)	33 (56.9)	0.248
Living area, *n* (%) Rural Urban	72 (50.3) 71 (49.7)	41 (48.2) 44 (51.8)	31 (53.4) 27 (46.6)	0.540
SD (yes), *n* (%)	18 (12.6)	10 (11.8)	8 (13.8)	0.918
EH (yes), *n* (%)	95 (66.4)	57 (67.1)	38 (65.5)	0.848
AF (yes), *n* (%)	26 (18.2)	13 (15.3)	13 (22.4)	0.278
CIHD (yes), *n* (%)	78 (54.8)	43 (50.6)	35 (60.3)	0.250
PVI (yes), *n* (%)	42 (29.4)	20 (23.5)	22 (37.9)	0.063
PF (yes), *n* (%)	22 (15.4)	13 (15.3)	9 (15.9)	0.971
COPD (yes), *n* (%)	38 (26.6)	19 (22.4)	19 (32.8)	0.167
Dyslipidemia (yes), *n* (%)	46 (32.2)	30 (35.3)	16 (27.6)	0.333
Diabetes (yes), *n* (%)	26 (18.2)	17 (20.0)	9 (15.5)	0.495
Surgical factors				
Side of the fracture, *n* (%) Left Right	58 (40.6) 85 (59.4)	34 (40.0) 51 (60.0)	24 (41.4) 34 (58.6)	0.869
ASA score, *n* (%) ˂III ≥III	49 (34.3) 94 (65.7)	30 (35.3) 55 (64.7)	19 (32.8) 39 (67.2)	0.754
Type of anesthesia, *n* (%) Spinal General	92 (64.3) 51 (35.7)	56 (65.9) 29 (34.1)	36 (62.1) 22 (37.9)	0.640
Days to surgery, 0–2 cut-off >2	71 (49.7) 72 (50.3)	48 (56.5) 37 (43.5)	23 (39.7) 35 (60.3)	0.048
LOHS (days), 0–8 cut-off >8	70 (49.0) 73 (51.0)	50 (58.8) 35 (41.2)	20 (34.5) 38 (65.5)	0.004
Duration of surgery (min), 0–58 cut-off >58	81 (56.6) 62 (43.4)	71 (83.5) 14 (16.5)	10 (17.2) 48 (82.8)	<0.0001
Laboratory data at admission				
Neutrophil count (×10^3^/µL), median (IQR)	6.99 (4.20)	7.89 (4.32)	6.77 (3.63)	0.059
Lymphocyte count (×10^3^/µL), median (IQR)	1.38 (0.88)	1.33 (0.75)	1.45 (1.04)	0.528
Monocyte count (×10^3^/µL), median (IQR)	0.71 (0.39)	0.70 (0.30)	0.73 (0.40)	0.177
PLT count (×10^3^/µL), median (IQR)	212 (94)	224 (98)	202 (80)	0.068
AST/ALT (>1, reference), median (IQR)	1.44 (0.59)	1.45 (0.61)	1.36 (0.59)	0.710
WBC (×10^3^/µL), median (IQR)	10.20 (4.20)	10.39 (4.53)	9.90 (3.41)	0.335
RBC (×10^6^/µL), median (IQR)	3.91 (1.26)	4.02 (1.22)	3.68 (1.06)	0.123
HGB (g/dL), median (IQR)	11.90 (3.38)	12.30 (3.20)	11.60 (3.04)	0.096
NLR (>6.57, cut-off), *n* (%)	46 (32.2)	29 (34.1)	17 (29.3)	0.546
PLR (>156.76, cut-off), *n* (%)	66 (46.2)	39 (45.9)	27 (46.6)	0.937
MLR (>0.54, cut-off), *n* (%)	69 (49.3)	37 (45.1)	32 (55.2)	0.241
SII (>1353.31, cut-off), *n* (%)	57 (39.9)	37 (43.5)	20 (34.5)	0.278
SIRI (>91.83, cut-off), *n* (%)	81 (56.6)	45 (52.9)	36 (62.1)	0.306
AISI (>834.86), *n* (%)	67 (46.9)	41 (48.2)	26 (44.8)	0.688
Laboratory data after surgery				
Neutrophil count (×10^3^/µL), median (IQR)	9.08 (5.52)	7.29 (4.35)	11.67 (4.55)	<0.0001
Lymphocyte count (×10^3^/µL), median (IQR)	1.17 (0.67)	1.36 (0.63)	0.93 (0.50)	<0.0001
Monocyte count (×10^3^/µL), mean ± SD	0.92 ± 0.41	0.81 ± 0.36	1.07 ± 0.42	<0.0001
PLT count (×10^3^/µL) median (IQR)	230 (96)	224 (97)	255.5 (70)	0.008
WBC (×10^3^/µL), median (IQR)	10.01 (5.81)	9.25 (4.14)	11.80 (6.74)	<0.0001
RBC (×10^6^/µL), median (IQR)	2.98 (0.78)	3.12 (0.83)	2.82 (0.55)	0.002
HGB (g/dL) mean ± SD	9.27 ± 1.69	9.60 ± 1.64	8.78 ± 1.65	0.004
NLR (>6.95, cut-off), *n* (%)	67 (46.9)	18 (21.2)	49 (84.5)	<0.0001
PLR (>186.13, cut-off), *n* (%)	76 (53.1)	30 (35.3)	46 (79.3)	<0.0001
MLR (>0.66, cut-off), *n* (%)	68 (48.2)	27 (32.1)	41 (71.9)	<0.0001
SII (>1970.47, cut-off), *n* (%)	76 (53.1)	22 (25.9)	54 (93.1)	<0.0001
SIRI (>167.54, cut-off), *n* (%)	78 (54.5)	27 (31.8)	51 (87.9)	<0.0001
AISI (>1857.45), *n* (%)	60 (42.0)	12 (14.1)	48 (82.8)	<0.0001

Abbreviations: SD—senile dementia; EH—Essential hypertension; AF—Atrial fibrillation; CIHD—Chronic ischemic heart disease; PVI—Peripheral venous insufficiency; COPD—Chronic obstructive pulmonary disease; ASA score—American Society of Anesthesiologists score; LOHS—Length of hospital stay; PLT—platelet; AST—aspartate-aminotransferase; ALT—alanine-transaminase; WBC—white blood count; RBC—red blood count; HGB—hemoglobin; NLR—neutrophil-lymphocyte ratio; PLR—platelet–lymphocyte ratio; MLR—monocyte–lymphocyte ratio; SII—systemic inflammation index; SIRI—systemic inflammation response index; AISI—aggregate inflammation systemic index. *p* value < 0.05 was attributed as statistically significant.

**Table 3:** Multivariate analysis of postoperative sterile inflammation.

Variable	Sterile Inflammation		*p*-Value
	OR	95% CI	
NLR Postoperative	2.91	1.28–6.61	<0.0001
PLR Postoperative	1.59	1.34–1.88	0.003
MLR Postoperative	1.45	1.18–1.78	0.005
SII Postoperative	1.02	1.001–1.03	0.039
SIRI Postoperative	1.04	1.01–1.07	0.028
AISI Postoperative	1.25	1.05–1.49	0.020
Days to surgery	1.34	1.04–1.72	0.009
Duration of surgery (min)	1.21	1.12–1.30	0.019
LOHS (days)	1.34	1.10–1.64	0.011

Abbreviations: NLR—neutrophil-lymphocyte ratio; PLR—platelet-lymphocyte ratio; MLR—monocyte-lymphocyte ratio; SII—systemic inflammation index; SIRI—systemic inflammation response index; AISI—aggregate inflammation systemic index; LOHS—length of hospital stay. *p* value < 0.05 was attributed as statistically significant.

## Data Availability

The data used in this study can be requested from the corresponding author.
